# Thriving in the City: An Urban Living Lab to Facilitate Recovery From Psychosis

**DOI:** 10.1111/eip.70257

**Published:** 2026-10-01

**Authors:** Lilith Abrahamyan Empson, Philippe Conus, Ola Söderström, Marc Winz, Sandra Vieira, Jérôme Favrod, Aurora Ruggeri, Jules Brischoux, Anastasie Jordan, Philippe Golay

**Affiliations:** ^1^ Treatment and Early Intervention in Psychosis Program (TIPP), Service of General Psychiatry, Department of Psychiatry Lausanne University Hospital CHUV Lausanne Switzerland; ^2^ Faculty of Biology and Medicine University of Lausanne Lausanne Switzerland; ^3^ Institute of Geography University of Neuchâtel Neuchâtel Switzerland; ^4^ Swiss Society of Social Psychiatry (So‐Psy.ch) Lausanne Switzerland; ^5^ La Source, School of Nursing Sciences HES‐SO University of Applied Sciences and Arts Lausanne Switzerland; ^6^ Institute of Psychology, Faculty of Social and Political Sciences University of Lausanne Lausanne Switzerland

**Keywords:** mental health recovery, psychosis, social determinants of health, structural determinants of health, urban health

## Abstract

**Aim:**

Protective factors and resources to recovery in urban milieus remain poorly understood, calling for user‐led initiatives to develop relevant interventions embedded in patients' daily‐life environments. Experience‐based co‐design is key to need‐based interventions in real‐life settings and calls for novel methodologies.

**Observation:**

This multi‐stakeholder (service users, psychiatrists, geographers, community actors and public authorities) initiative aims to identify key elements of an ‘urban recovery milieu for psychosis’ in Lausanne, Switzerland based on a Living Lab approach. Beyond questions of usage, feasibility and acceptability, we will identify which types of interventions are more useful for which group of patients. This pilot phase may pave the way towards a future randomized control trial and the definition of the most appropriate sample size for it.

**Results:**

Qualitative data are collected through field notes, audio‐recorded walk‐alongs, workshops and focus groups with care teams and participants. The quantitative analysis is based on observational and correlational study design. We will assess how 55 patients of two psychosis‐specialized cohorts use the urban interventions in their day‐to‐day practices and monitor psychometric variables before and after the experimentation. The data stems from tailor‐made questionnaires (City avoidance questionnaire, Urban remediation questionnaire, etc.), clinical rating scales (ASI, RAS, TEPS, ISMI and CRDPS) and routine follow‐up procedures (SOFAS, GAF, CGI, WHOQOL and PAS).

**Discussion:**

Additionally, insights from this study will inform mental health plan on municipal level and be upscaled for broader public benefits.

## 1 Introduction

This paper presents the design of an ongoing study (1 March 2023 to 31 August 2026) that seeks to address the gaps of previous research by developing a context‐specific strategy co‐designed with first episode psychosis (FEP) patients and diverse stakeholders from their wider social environment. Building on emerging perspectives that connect personal resilience with broader socio‐material environments, and more specifically urban settings, we argue for a shift from individual‐focused models towards approaches that promote social and material changes needed to make the urban environment a supportive setting where individuals' personal resilience can fully unfold. Finally, we introduce a Living Lab‐based participatory research approach designed to co‐create practical, small‐scale urban interventions with people with lived experience and key community stakeholders to foster recovery from psychosis.

Whilst recovery has become a mainstream paradigm in mental health care across much of the Western world (Norton 2022), it lacks a universally accepted definition and is often examined through the related construct of resilience. Resilience has been positively associated with sustained full recovery, community functioning (Wambua et al. 2020) and quality of life amongst individuals with schizophrenia (Torgalsbøen 2012; Torgalsbøen et al. 2018). It also enables young people with psychosis to overcome major psychosocial challenges (Henderson and Cock 2015) and may influence the transition from an at‐risk state to psychosis (Rossi et al. 2017), underscoring the importance of environmental factors in recovery. As vulnerabilities evolve across changing life circumstances, individuals may continue to adapt positively despite significant adversity (Luthar et al. 2000; Rutter 2012). Furthermore, resilience can be fostered when environments facilitate access to supportive resources (Stainton et al. 2019; Ungar 2019). Together, these findings suggest that recovery‐oriented care should extend beyond an individual‐centred view of resilience to incorporate broader social environments and place greater emphasis on protective rather than risk factors.

Although resilience‐enhancing factors and their interactions are central to recovery‐oriented interventions (Fritz et al. 2018), contemporary psychiatric research continues to focus predominantly on risk factors (Serra‐Arumí et al. 2024), both when it comes to social determinants (Kirkbride et al. 2024) and neural processes involved in adaptation to adversity. Yet, evidence that brain regions implicated in resilience overlap with those associated with the increased risk of schizophrenia following early‐life urban exposure (Akdeniz et al. 2014; Besteher et al. 2017; Haddad et al. 2015; Lederbogen et al. 2011; Tost et al. 2015) highlights a more complex interplay between the brain and the wider social environment. Accordingly, Holz et al. (2020) advocate a shift from vulnerability‐based models towards a protective‐factor framework centred on neural resilience, through which therapeutic interventions and supportive social environments may exert neurobiological effects. This perspective is supported by evidence demonstrating the protective effects of supportive social networks, from close interpersonal relationships to neighbourhood‐level interventions (Herrman et al. 2011; Kirkbride et al. 2024; Leff 1997).

Despite growing recognition of the social dimensions of recovery, classical psychiatric settings continue to emphasize personal resilience, autonomy and individual responsibility, particularly in relation to engagement in treatment, symptom management and adherence to care. Whilst this focus has contributed to the development of person‐centred mental health practices, it may also obscure the extent to which recovery is shaped—and at times constrained—by broader structural factors, including public policies, social services, physical environments and material living conditions (Slade et al. 2008). Indeed, recovery is increasingly understood as an inherently social process (Leamy et al. 2011; Topor et al. 2011), embedded in specific geographical contexts (Söderström et al. 2017) and grounded in everyday community life (Davidson 2009; Ornelas et al. 2014; Ware et al. 2007). From this perspective, recovery can be conceived as an improved person–environment fit (Rappaport 1987), sustained through the exchange of resources amongst individuals and communities (Trickett 2009). Accordingly, Leamy et al. (2011) argue that recovery frameworks centred on individual meaning and experience may underemphasize the wider socio‐environmental context, whereas Topor et al. (2011) highlight the importance of social relationships, material conditions such as housing, employment and income, and access to knowledge about recovery.

This calls for a reconfiguration of our understanding of mental health and illness as emerging from the daily experience of selfhood and ‘wider, more‐than‐human compositions and configurations’ of the environment: a conceptual framework known as EnvironMental health (Malafouris et al. 2025). In contrast with classical understanding of environmental health, primarily concerned with environmental hazards, pollutants or risk exposure (Briggs 2003; Frumkin 2016), this theoretical concept explores how material affordances and the environment shape psychological states and disorders at a fundamental conceptual level. It reframes mental health as not merely internal brain processes but part of a continuous system with the physical, social and material surroundings by which it is deeply co‐constituted. Such perspective resonates with a relational understanding of health and place in human geography, which conceptualizes health not as an inherent individual trait but as an emergent property of dynamic interactions and connections between people, places and materials (Conradson 2005; Cummins et al. 2007). Since the 1990s, this line of inquiry has been enriched through the development of concepts such as therapeutic landscapes (Gesler 1992), landscapes of recovery (Milligan and Wiles 2010) and enabling places (Duff 2012) which shifts the focus to the places, processes and networks that constitute well‐being, arguing that bodies and environments mutually co‐produce health outcomes. Such work underscores the role of place‐making and everyday activities as vital resources mental health (Doroud et al. 2018; Duff 2012).

This further underlines the need for an articulation between lived experience informed perspectives and a thorough scrutiny of the role the environments by which the experience of the self and social processes is shaped. Urban environments exemplify this issue, as cities are complex environments where well‐documented risk factors for psychosis (Abrahamyan Empson et al. 2020; Krabbendam et al. 2021) combine with better access to care and resources for social, economic and cultural integration. Given that most of the world's population already lives in cities and that urbanization is expected to grow at an even faster pace, interactions between urbanization and mental health and, by extension, recovery from psychosis in urban environments are gaining increasing scientific interest (D'Acci 2020; Okkels et al. 2018). This calls for a thorough scrutiny of material aspects of cities and their social fabric in relation to FEP patients' urban practices and recovery.

This study builds on a previous Swiss‐based project; we explored how the city of Lausanne generates disruptive or protective milieus in the life trajectories of FEP patients and controls (Conus et al. 2019; Söderström et al. 2016, 2017). A significant proportion of our early intervention cohort displayed *city avoidance*: a drastic decrease in city attendance after their first episode of psychosis. In addition, they seemed to derive fewer benefits than controls from the city's restorative resources (Roe and McCay 2021), such as parks or green spaces; a phenomenon we termed *city anhedonia*. Given that both *city avoidance* and *city anhedonia* may potentially hinder access to urban resources for recovery and be self‐perpetuating, we have previously called for a creation of city‐specific recovery‐oriented interventions: an approach we have termed *Urban Remediation* (Baumann et al. 2020). We hypothesized that alleviating *city avoidance* and *city anhedonia* could promote recovery by improving patients' engagement with urban resources. However, the term remediation heavily relies on promoting patients' resilience. The objective of the current study is therefore not simply to help individuals to adapt to urban environments but to understand how environments themselves can be shaped to support recovery. This project's central hypothesis is that access to urban resources constitutes a key determinant of recovery. The guiding research question is therefore: *How can an urban recovery milieu for psychosis be fostered*?

Bringing together patients, peer practitioners, mental health professionals, civil society organizations, municipal authorities and researchers, the project is grounded in participatory approaches that have demonstrated the value of partnerships in designing recovery‐oriented interventions. A growing body of research supports that user‐driven services, focused on reducing internalized stigma and return to full citizenship for people recovering from mental illness, are valuable in improving the outcome of therapies and empowering persons suffering from psychosis (Bond et al. 2004; Drake and Whitley 2014; Leamy et al. 2011; Lequin et al. 2023; Nelson et al. 2014; Pelletier et al. 2015). This emphasis is reinforced by the right to full citizenship enshrined in the United Nations Convention on the Rights of Persons with Disabilities,^1^ highlighting the importance of meaningful involvement of people with lived experience in designing the services and policies that affect them.^2^ Accordingly, shared decision‐making in policy and programme design, implementation and evaluation is recognized as a cornerstone of genuine collaboration and co‐production in effective recovery‐oriented services (Norton 2025; Ornelas et al. 2019; Sundet et al. 2020). Evidence further suggests that user‐operated services and peer support benefit both recipients, through exposure to positive role models (Wallot 2019), and providers, through enhanced confidence and self‐esteem associated with improved recovery outcomes (Corrigan 2006; Jambawo et al. 2024; Resnick and Rosenheck 2008). More broadly, fostering connectedness, interdependence within communities and relationships beyond the mental health system has been consistently linked to more positive recovery trajectories (Lloyd et al. 2006; Ornelas et al. 2014; Ware et al. 2007).

## 2 Methods and Analysis

### 2.1 A Living Lab Approach to Urban Mental Health

This project is grounded in a participatory action research (PAR) design. More specifically, it follows a Living Lab approach.

Living Lab projects are characterized by quasi‐experimental approaches and user involvement, fore‐grounding co‐creation (Franz et al. 2015; Puerari et al. 2018; Evans 2019). Whilst Living Lab approaches originate in the context of business and marketing strategies—where they were used to optimize product development processes by involving clients and future users—they later quickly spread to other realms with a problem‐solving approach. Living labs can also be seen as a methodology, an environment, a system or an innovation approach (Bergvall‐Kåreborn et al. 2009), resulting in varying definitions depending on the setting, context or aim of the research (Hossain et al. 2019).

In recent years, Living Labs have been increasingly used in urban contexts (Marvin et al. 2018), where traditional—top‐down and non‐participatory—planning approaches show their limits (Evans et al. 2018; Voytenko et al. 2016). In this context, empirical data involving local and experiential knowledge is of crucial importance, in contrast with simply transposing ready‐made urban models and urban interventions from one place to another. Concurrently, Living Labs are increasingly regarded as a promising approach in health research (Borda et al. 2024) gaining traction as a method for the successful implementation of innovations in healthcare delivery (Figueiredo et al. 2024; Watson et al. 2026; Zipfel et al. 2022). Living Labs are being established to study and enhance mental well‐being at University College London^3^ and the Universitat Autònoma de Barcelona,^4^ with a focus on youth mental health promotion, as demonstrated by Oxford University, alongside the creation of a network within the ALIVE National Centre for Mental Health Research Translation in Australia^5^ to integrate people with lived experience of mental ill‐health into research design and the co‐creation of services, models of care and digital tools for mental health. Richardson et al. (2021) have proposed the ‘Brain Health Living Labs’ as user‐centred, iterative ecosystems, integrating concurrent clinical care, research and innovation processes in geriatric contexts, alongside the development of dementia‐related issues. To the best of our knowledge, no study has yet developed a living lab approach in the context of city–psychosis nexus.

Living Labs can take different forms in practice (Watson et al. 2026). As a research approach, they most commonly bring together four types of stakeholders—citizens or users, professionals, academics and public authorities—into a participatory process aimed at generating knowledge and fostering (social) innovation (Franz et al. 2015; Kim et al. 2019; Palmer et al. 2023; Puerari et al. 2018). This process generally follows four iterative and reflexive phases: (i) exploration, (ii) co‐elaboration or co‐creation, (iii) experimentation and (iv) implementation and upscaling (Ballon et al. 2018; Malmberg et al. 2017; Van Den Kieboom et al. 2019). Each phase of this incremental process informs and shapes the following.

### 2.2 Patients

This study is embedded in the outpatient clinic of Lausanne University hospital, Switzerland. Study participants will be residents or users of the Living Lab's neighbourhood (zip codes: 1003, 1004, 1005, 1006, 1007 and 1009) and recruited from two specialized psychosis cohorts to better represent the span of recovery trajectories after a FEP. For cohort specifics and inclusion/exclusion criteria see Tables 1 and 2. By purposefully selecting participants as diverse as possible across key dimensions, in our case, city attendance based on time they spent in cities (City avoidance questionnaire, Abrahamyan Empson et al. 2025) and stages of recovery, based on RAS Scale, we will seek to identify both shared patterns and context‐specific differences across heterogeneous cases. This approach prioritizes diversity over representativeness to better understand the complexity of the phenomenon under study.

**TABLE 1:** Cohort description.

	Cohort specifics
Treatment and early Intervention in Psychosis Programme (TIPP)	– Intensive, multidisciplinary outpatient care for FEP in Lausanne catchment area. – The criteria for admission ✓ age between 18 and 35, psychotic symptoms exceeding the threshold of psychosis according to the CAARMS and ✓ not having benefited from an anti‐psychotic treatment for more than 6 months. *all TIPP patients are assessed every 6 months for the entire duration of the programme (36 months) to gather information on sociodemographic (migrant status, SES, living arrangements, etc.), premorbid, baseline and outcome characteristics related to a wide range of clinical features (DUP, negative/affective symptom severity, etc.), comorbidities (substance use), treatment responses (symptom severity, antipsychotic dose, concurrent psychological therapies, etc.), and symptomatic and functional outcomes. This existing clinical data will be used retrospectively in our study (CER‐VD #2020‐00272).
Minkowski care Programme	Patients suffering from different forms of longer‐lasting psychoses who ✓ either cannot be included in the TIPP (e.g., psychosis onset after 35) or ✓ have already completed their follow up within the TIPP but are still in need of care.

**TABLE 2:** Inclusion and exclusion criteria.

Inclusion criteria	Exclusion criteria
Follow‐up/care in the TIPP/Minkowski programme Patients aged between 18 and 45 years old to minimize age‐related trends Patients suffering from different forms of psychosis (F20–29) based on ICD‐10 (International Classification of Diseases) Living or using the Living Lab neighbourhood (zip codes: 1003, 1004, 1005, 1006, 1007 and 1009) Informed consent	Age over 45 Absence of diagnosis of psychosis according to ICD‐10 Patients not living or using the Living Lab neighbourhood. Patients whose active participation in the study is judged as a major risk of relapse by their care team Lack of informed consent Insufficient knowledge of French language

### 2.3 Data Collection and Analysis

Data collection and analysis combine qualitative and quantitative methods (Table 3) bringing insights from psychiatry and human geography, embedded within the incremental design of the project where each stage informs the following one. Data are regularly presented for validation and complementary inputs to key stakeholders and experts to reflect on scientific and practice relevance throughout the project.

**TABLE 3:** Synthesis of mixed‐methods and preliminary insights for each phase of the study.

	Qualitative	Quantitative	Preliminary insights
Exploration	Audio‐recorded 40 two hours systematic transects – semi‐structured immersive interviews – field notes – photos		✓ Systematic participatory mapping of obstacles and resources ✓ Preliminary list of candidate interventions
Co‐creation	Two participatory design workshops Two focus groups – Inductive analysis of workshop material		✓ Short list of up to six interventions
Experimentation	Two rounds of 6–10 semi‐structured interviews with purposeful sampling of patients amongst 55 recruited at T1 and T2	Tailor‐made questionnaires ✓ City avoidance questionnaire ✓ Urban remediation questionnaire ✓ Intervention evaluation questionnaire Psychometric assessment ✓ Self‐assessment questionnaires (*N* = 55) ✓ Clinical rating scales (*N* = 55)	Preliminary insights on ✓ acceptability and feasibility ✓ correlations between recovery and usage of the urban interventions ✓ relevance of each specific interventions for the sub‐groups of patients according to their level of city‐avoidance and recovery ✓ outcomes of interest for Phase 4 calculation of a sample size for a randomized study
Implementation and up‐scaling	No data collection planned		Roadmap to scaling from neighbourhood to city level

#### 2.3.1 Phase 1—Exploration: Participatory Mapping

The exploration phase consists of the analysis of the everyday urban context of patients' lives. It aims to understand their practices, to identify and locate urban resources for wellbeing and recovery, and what hinders access to them. They also allow for identify relevant stakeholders for the upcoming steps. In our study, this phase takes the form of a participatory mapping, where go‐along interviews serve as the primary method of data collection, as they are particularly effective for examining the interplay between health and place (Carpiano 2009). The interviews take place in three pairs of researcher and participant, whilst one researcher additionally partners with an expert by experience. All interviews are supported by a semi‐structured guide and are audio recorded. Participants are provided with cameras to document meaningful urban elements. Four 1‐h itineraries were developed to capture a range of urban settings with flexibility to pause or adjust the pace, if needed. Each walk begins with a short briefing on the route and aims and ends with an informal discussion, where participants are asked to annotate printed maps with comments or sketches. For a systematic assessment, the routes are predefined and guided by the research team. As a result, the dataset for this phase includes audio recordings of the go‐along interviews (*N* = 36), participant photographs, annotated maps and the group discussion.

The research team transcribed all interviews and imported the text, alongside participant photographs, into MAXQDA. In the process each photograph is linked to corresponding verbatim passages; this integration enriches the narrative data and aligns with the use of the photo cameras as prompts that facilitate participant engagement with their surroundings. The transcribed interviews are then coded following a thematic analysis procedure (Ahmed et al. 2025). Coding relies on the social, material and affective dimensions of enabling places (Duff 2012), and the recovery‐oriented, place‐based relations categories of being, doing, becoming and belonging (Doroud et al. 2018) on the one side. On the other, whilst these frameworks provide initial categories, additional codes are added based on the data, as the analysis progresses. Thus, the study employs an abductive thematic analysis, a method that uses existing theories and literature concurrently with raw data to understand underlying patterns and narratives (Thompson 2022, 1411).

#### 2.3.2 Phase 2—Co‐Creation: Designing the Interventions

*Co‐creation* refers to the collective and collaborative process of elaborating strategies to promote an environment more prone for recovery through an iterative process. The results of the participatory mapping are reported to inter‐ and trans‐disciplinary panels and participants in a dedicated workshop to co‐identify needs and co‐elaborate relevant strategies to be tested. Throughout the course of the project, relevant stakeholders gradually come on board the abovementioned panel, and preliminary findings are reported back to it to refine subsequent steps. A comprehensive description of the co‐creation process lies beyond the scope of the present article and will be reported in a separate publication. By the end of this phase, the following interventions were selected for experimental implementation within the study neighbourhood:

1. Provision of training sessions on efficient and appropriate ways to interact with individuals experiencing psychosis to be delivered to bus controllers, surveillance personnel of three museums and staff at the city's major library.
2. Provision of a digital material on mental health and cities and hand‐outs on appropriate ways to interact with individuals experiencing psychosis in cafés of the neighbourhood.
3. Provision of eight guided museum tours, each combined with creative workshops involving study participants.
4. Establishment of a dedicated mental health corner at the city's major library.
5. Development of a sensory, immersive pathway in one of the parks located in the city centre.
6. Provision of 10 walking groups connecting the intervention sites, incorporating opportunities for socialization and stress management in urban environments, co‐developed with peer practitioners.

These interventions were selected from a broader pool of proposals, as our transdisciplinary panel identified them as the most relevant to the overarching objectives of the study. It has been hypothesized, that reducing stigma, enhancing social participation and a better stress management will reduce city avoidance and city anhedonia (primary outcomes), and lead to a greater sense of personal recovery and better quality of life (secondary outcomes). The selection of these interventions also informed the choice of psychometric measures employed in the study (see Table 4) to conduct an exploratory analysis. The latter shall shed light on underlying hypothetical mechanisms of change (reducing internalized stigma, enhancing anticipatory and consummatory pleasure, better urban stress management) and possible intergroup and intra‐ individual differences over different time points regarding psychopathological and demographic characteristics.

**TABLE 4:** Synthesis of quantitative data collection according to different time points.

Timepoints		T1	T2
Tailor‐made questionnaires			
1.	City avoidance	X	X
	Urban remediation questionnaire	X	X
	Intervention evaluation questionnaire		X
2.	Recovery Assessment Scale (RAS)	X	X
3.	Aberrant Salience Inventory (ASI)	X	X
4.	Temporal Experience of Pleasure Scale (TEPS)	X	X
5.	Internalized Stigma Scale (ISMI)	X	X
Medical records based data			
1.	Clinician Rated Dimensions of Psychosis Symptom Severity (CRDPS)	X	X
2.	Social and occupational functioning assessment scale (SOFAS)	X	X
3.	Global assessment of functioning (GAF)	X	X
4.	Clinical global impression (CGI)	X	X
5.	World Health Organization Quality Of Life assessment (WHOQOL)	X	X
6.	Premorbid adjustment scale (PAS)	X	

Abbreviations: T1 = baseline; T2 = 8 months after the beginning of experimentation phase.

#### 2.3.3 Phase 3—Experimentation: Testing and Evaluating

*Experimentation* comprises testing and evaluating the solutions in a real‐life setting. We experiment with the co‐elaborated interventions in the predefined catchment area for 8 months. Both qualitative and quantitative methods are used to assess feasibility and gather insights on usage and relevance of the interventions, with the data collection completed by March 2026.

##### 2.3.3.1 Qualitative Methods

Qualitative data is collected through focus groups with participants selected through convenience sampling and observations and reports collected by the care team during usual treatment. Focus groups evolved around participants' and first line caregivers' perspectives on interventions' acceptability, feasibility, perceived usefulness, timeliness and relevance to daily care. Audio‐recorded material was then transcribed and coded following thematic analysis (Ahmed et al. 2025). Analytical rigour was maintained through iterative team discussions involving a lived‐experience researcher.

##### 2.3.3.2 Quantitative Methods

We have recruited 55 patients, who will receive treatment as usual within respective treatment programmes. Our aim is to assess the way patients use the urban interventions in their day‐to‐day practices within our Urban Lab and to monitor psychometric variables over the course of 8 months. This will allow us to explore (i) *acceptability and feasibility* of the interventions and establish (ii) *correlations between recovery and usage* of the urban interventions. In addition, using ‘maximum variation sampling’ (Patton 2002; Silva et al. 2023) allows us to (iii) *identify sub‐groups of patients according to their level of city‐avoidance and recovery, and thus explore relevant urban interventions for the diverse groups of patients*.

We recruited 55 patients receiving treatment as usual within their respective treatment programmes. The quantitative component is a pilot repeated‐measures study designed to assess the feasibility and acceptability of the urban interventions and to explore whether intervention use is associated with change in recovery‐related outcomes over 8 months. Quantitative assessments are conducted at baseline and at the end of the test period. The primary analyses will consist of (1) descriptive statistics summarizing intervention uptake, attendance and acceptability/feasibility indicators; and (2) within‐subject analyses of change over time in predefined primary outcomes (city avoidance and city anhedonia) and secondary outcomes (personal recovery and quality of life). Secondary exploratory analyses will examine whether the degree of intervention use/city attendance is associated with changes in these outcomes. A limited set of clinically relevant variables routinely collected in care, including DUP, treatment adherence, symptom severity, antipsychotic dose, substance use, migrant status and SES, will be considered as adjustment variables where available and depending on sample size. In addition, exploratory subgroup analyses will examine whether patterns differ according to baseline levels of city avoidance and recovery. Given the pilot nature of the study and modest sample size, these analyses are hypothesis‐generating rather than confirmatory. *A package of tailor‐made questionnaires* will consist of:

- *City‐avoidance questionnaire* (Conus et al. 2019) captures the way patients use and experience the city. The questionnaire consists of three main parts to gather information on relevant sociodemographic characteristics, participants' city attendance (frequency and duration), their perception of various specific places of the city and sensory and interactional dimensions of city living.
- The *Urban remediation questionnaire* addresses the specific needs of patients regarding a set of recurrent problems and their perception of the neighbourhood.
- The *Intervention evaluation questionnaire* relates more specifically to the usage of the implemented urban interventions.

All questionnaires were approved by the local Ethics Committee before implementation. Quantitative evaluation takes place before onboarding of patients, as well as after the test period.

*Psychometric assessment* will juxtapose elements of theory (e.g., sensory overload, stigma, anhedonia and recovery stage) with potential changes in urban practices and recovery (see Table 4). Beyond questions of feasibility and acceptability of the implemented interventions, we will be able to further refine outcomes of interest and variables linked to changes, as well as identify which types of interventions are more useful for which group of patients. This pilot phase may pave the way towards a future randomized controlled trial and the definition of the most appropriate sample size for it.

#### 2.3.4 Phase 4—Implementation and Upscaling: From the Living Lab to the Municipal Level

*Upscaling*: Implementation of research findings in organizational and quality of care setting remains a major challenge. A living lab approach enhances chances for healthcare innovations to be implemented, as well as it identified as series of factors—leadership, involvement, timing, openness, organizational support and ownership—contributing to this success (Zipfel et al. 2022). The unique nature and ambition of the project—spanning across psychiatric care and public health—renders this endeavour even more complex. Thus, we aim to achieve upscaling through the development of a municipal mental health plan, involving relevant municipal actors in the City of Lausanne (public health, housing, urban planning, mobility, public spaces, culture and social services departments).

## 3 Conclusions

The use of real‐world methodologies and the mobilization of different stakeholders is an important step towards a more fine‐grained understanding of urban resources and obstacles to recovery and the creation of need‐based prototype interventions for effective user‐based changes and the implementation of sustainable solutions in real world settings. This pilot study will help us define the feasibility of shortlisted interventions and gain some insights into hypothetical mechanisms by which urban environments may foster recovery, alongside identifying pitfalls and possible risks before a randomized‐controlled study. Prototype solutions can be scaled up at a municipal level for more inclusive cities, beneficial to the community as a whole.

## Funding

This work was supported by the Humanities and Social Sciences Division of the Swiss National Science Foundation (Grant No. 212633).

## Ethics Statement

The study received approval from the local Ethics committee (CER‐VD, reference no. 2022‐02257) and permission to reuse previously collected data from routine follow‐up procedures under a separate approval (CER‐VD, reference no. 2020‐00272).

## Consent

All participants provided written informed consent prior to participation in the study. Consent procedures were conducted in accordance with ethical guidelines for research involving potentially vulnerable populations, with particular attention to capacity to consent, voluntariness and comprehension. Participants were fully informed about the study aims, Living Lab and co‐design activities, data collection procedures, potential risks and benefits and data protection measures, including anonymization. Consent was obtained for the collection, analysis and publication of anonymized quantitative data and qualitative materials, including interview excerpts and outputs from participatory workshops. Participants were informed of their right to withdraw from the study at any time without any consequences.

## Data Availability

In accordance with local Ethics Committee requirements, fully anonymized quantified data will be shared upon the publication of respective outputs after ensuring study participants full consent and preservation of confidentiality on zenodo archive (https://zenodo.org/).

## Endnotes

^1^

https://www.un.org/development/desa/disabilities/.

^2^

https://academic.oup.com/california‐scholarship‐online/book/18906.

^3^

Designing healthier places | Portico Magazine | UCL.

^4^

The UAB launches the Living Lab in Mental Health – Universitat Autònoma de Barcelona – UAB Barcelona.

^5^

Co‐Design Living Labs Network – The Alive National Centre.
